# From TELOCYTES to TELOCYTOPATHIES. Do Recently Described Interstitial Cells Play a Role in Female Idiopathic Infertility?

**DOI:** 10.3390/medicina56120688

**Published:** 2020-12-11

**Authors:** Martin Klein, Lenka Lapides, Denisa Fecmanova, Ivan Varga

**Affiliations:** 1 Faculty of Medicine, Institute of Histology and Embryology, Comenius University in Bratislava, 81372 Bratislava, Slovakia; martin.klein@fmed.uniba.sk; 2 ISCARE, Reproduction Clinic, Gyneacology & Urology, 82109 Bratislava, Slovakia; lenka.lapides@iscare.sk; 3 First Department of Gynecology and Obstetrics, Faculty of Medicine, Comenius University in Bratislava and University Hospital, 81372 Bratislava, Slovakia; denisa.fecmanova.df@gmail.com

The invention of such state-of-the art microscopic techniques like atomic force microscopy with a resolution of fractions of a nanometer, and the overall current level of knowledge in morphological disciplines have brought about a widespread impression, that identifying a new cell population in the 21st century is highly unlikely. Yet, a research team from Bucharest, Romania led by professor Popescu (1944–2015) shattered this notion in 2005, by discovering a new cell population called interstitial Cajal-like cells, later renamed to telocytes.

Telocytes are interstitial cells, which have been described in almost all organs of the human body, and also in many non-human animal species. Their functions have been characterized as being hugely heterogeneous, from cell-to-cell signalling, immune surveillance, stem cell nursing or even hormone-sensing. Apart from their presupposed physiological functions, they have also been described as important pathogenetic triggers of many different disease entities. Based on the number of diseases, the heterogeneity of organs in which they develop, and a putative yet key role telocytes possibly play in their aetiopathogenesis, we proposed to call these conditions “telocytopathies”. The main puzzle, which has to be resolved in future research regarding “telocytopathies” is the problem of correlation vs. causation. It is so because most of the papers discussing telocytes in disease development, mention two main pathogenetic factors—functional derangements and quantitative loss of telocytes. However, it is considerably obscure whether telocytes are functionally and quantitatively diminished intrinsically, and subsequently cause a disease, or there is some unknown variable or factor which causes the disruption of the whole cellular microenvironment of a given organ, leading to disease, in which the observed telocyte loss and functional defects are solely a consequence of an overall microenvironmental disruption [1].

As already mentioned, telocytes have been described in almost all organs of the human body; in some only marginally, while in others, they have been researched extensively. The latter category includes the organs of the female reproductive system. One of the main fields of interest in the study of telocytes in the organs of the female reproductive system is their role in the pathogenesis of infertility. 

One of the obvious causes coming under the category of “female factors” are different pathological changes of the uterine tubes. The best examples are endometriosis, postoperative complications of abdominal surgeries, or post-infectious changes. However, some deleterious effects on the delicate regulation of tubal function can be subtle and thus less obvious, which can lead to the diagnosis of idiopathic infertility, but in fact there is a cause yet to be recognized. Pathological changes in telocytes of the uterine tube can play a role in both scenarios. Tubal telocytes are located in close vicinity to smooth muscle cells and establish cell-to-cell contacts with them. They make contacts with immune cells, and also serve as hormonal sensors. It is known that tubal motility is hormone-dependent, so under normal conditions, telocytes possibly regulate this function as well. Therefore, telocytes can be viewed as cells with an important role in the structural and functional integrity of the tubal cellular microenvironment. An original paper by Yang et al. (2015) using a rat oviduct model of endometriosis, discussed an interesting finding, that telocytes are excessively fragile in the presence of noxious factors, compared to other stromal cells [2]. Even in the absence of prominent tubal pathology, a subtle dysregulation of highly complex immune regulation necessary for successful conception, eventually leading to idiopathic infertility, is mediated by multiple factors, mainly of the adaptive immune system. It is possible though, that telocytes might be the “last piece of the puzzle” which will elucidate the complex pathophysiology of female infertility, especially the unexplained subcategory.

The organ of utmost importance in terms of infertility is the uterus. Like tubal telocytes, telocytes in the uterus have been implicated in both explainable and idiopathic infertility. One of the most common uterine factors associated with infertility are leiomyomas or fibroids. We performed an immunohistochemical study which demonstrated the absence of telocytes in the neoplasm, and hypothesised their possible roles in this process, from hormone-sensing to telocytes influence on angiogenesis [3]. A successful implantation of an early embryo into the secretory endometrium demands a unique fine-tuning of the endometrial immune reactions which will allow a blastocyst to escape the immune system recognition as non-self, necessarily leading to embryo rejection. This process is influenced by various factors from embryonic to maternal. From the early embryonic perspective, its syncytiotrofoblast produces many important substances, most notably human chorionic gonadotropin (hCG), which is a potent immune regulator. From the maternal side, many different populations of immune cells play a role, most notably macrophages, which are the second most numerous decidual immune cell population and Natural Killer cells (NK cells) which are quantitatively the most dominant in the decidua during the first trimester. They have many different roles, but both are crucial in the regulation of implantation and placentation. Equally important are embryonic/foetal macrophages called Hofbauer cells which, among other functions, protect the embryo/foetus from being rejected by the immune system. The active role of uterine telocytes in immune regulation and surveillance has been experimentally demonstrated in vitro. Telocytes were able to activate macrophages via paracrine signalling pathways [4].

Another important aspect of successful embryo implantation is the proper decidualisation of the endometrium, involving proliferation and differentiation of stromal cells into decidual cells. An experimental co-culturing of telocytes with endometrial stromal cells revealed the formation of cell-to-cell contacts and this interaction enhanced proliferative, migratory and adhesive capacity of endometrial stromal cells. Even though this experiment was focused on elucidation of the pathogenesis of endometriosis, these findings can perhaps also be applied to endometrial stromal cells undergoing decidualisation [5].

Telocytes are a peculiar cell population with many presupposed physiological functions, as well as multifaceted involvement in the pathogenesis of a broad spectrum of diseases—“telocytopathies”. Their role in the pathogenesis of idiopathic infertility and also other pathologies of the female reproductive system is still highly speculative. Nevertheless, when we consider their discussed role in fine regulation of immune functions, cell-to-cell signalling and others, the future directions of telocytes research are clearly marked. We think that the principal future perspective is to resolve the issue of “telocytopathies”, in which the researchers will have to filter out those conditions that are truly caused by telocyte derangement from those which are not. This will possibly change the status of many “idiopathic” diseases regardless of the organ in question.
